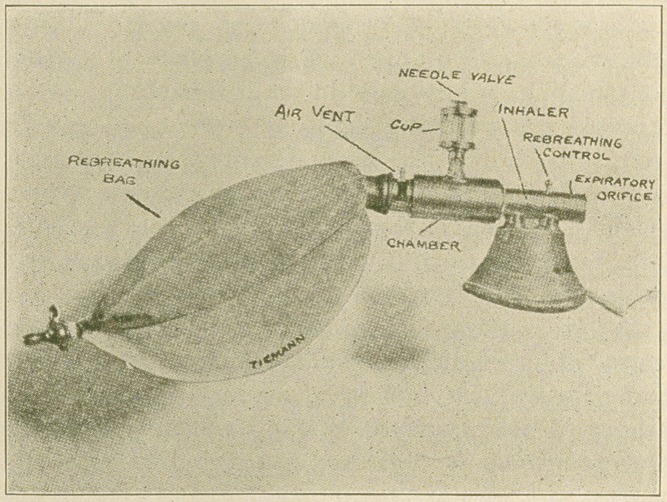# A New Apparatus for Administering and Warming General Anesthetics and New Methods of Administration

**Published:** 1912-04-15

**Authors:** Raymond C. Coburn

**Affiliations:** New York


### A NEW APPARATUS FOR ADMINISTERING AND WARMING GENERAL ANESTHETICS AND NEW METHODS OF ADMINISTRATION.

BY RAYMOND C. COBURN, M.A., M.D., NEW YORK.

I have devised a new apparatus primarily for the administration of nitrous oxid and oxygen, the chief aim being simplicity, utility and portability. I have also perfected two new methods of administering: ether by the closed drop method, and the warm vapor method, whereby the vapor of all anesthetics by all methods is warmed to body temperature at the time it is inhaled. Several other original and practical features will appear in the following description:

### APPARATUS.

A light stand is provided for holding two cylinders each of nitrous oxid and of oxygen. The supporting standard has slip, instead of threaded, connections with the base and the top bearing the cylinders so as to be quickly assembled and dissembled. Small rubber tubing leads from the top of the stand to an 8-liter gas bag, the neck of which is attached to a metal fitting, which carries a shut-off and air vent operated together, and is attached to one end of the anesthetic chamber. This opening of the chamber is quite large and through it the necessary gauze may be easily and quickly placed within. As the chamber is ample in size it is never packed, but just loosely filled, with coarse gauze. To the top of this chamber is attached the anesthetic cup, which has a needle-point valve controlling the flow of the liquid anesthetic to the chamber containing the gauze. As the anesthetic drops from the cup it can be seen plainly and the amount accurately regulated by the needle-point valve. The liquid anesthetic is added by a mechanical drop method, thus insuring it to be regular and constant, and at the same time the flow is in sight and under complete control of the anesthetist.

The other end of the chamber is attached to the inhaler, which has an expiratory orifice that is open when the exhalations are to escape, and closed when there is rebreathing. The inhaler carries an inner tube in which are two light and delicately constructed valves, so arranged that they are thrown out of action when there is rebreathing, and, when the exhalations are to escape, one valve prevents breathing back into the bag during expiration, and the other prevents any air from being inspired through the expiratory orifice during inspiration, the change from one form to the other being easily and quickly made by simply turning the knob through an angle of 90 degrees. The expiratory orifice always directs the exhalations away from the patient's face and field of operation. Through this orifice, the expiratory valve can be plainly seen, and its movement in the open method always shows whether the patient is actually breathing, while the movements of the bag furnish this valuable information in the closed method.

To the other end of the inhaler is attached the face mask, which is made of transparent celluloid or of metal, and of such a shape that when the rubber hood is inflated it fits the face very accurately.

The cup, chamber and bag, when in position, ordinarily extend back over the patient's head, but by reversing the mask on the inhaler, they may be extended over the patient's chest. The cup can always be maintained in an upright position, no matter what may be the position of the patient's face, by turning the chamber on its connection with the inhaler. When the ether attachment is not needed, the cup and chamber may be removed and the bag fitting attached direct to the inhaler.

A small and light electric heater, with a flexible cord which may be connected to any lamp socket (either direct or alternating current), is attached by spring clamps to the chamber, and it thoroughly warms all the anesthetic vapor. This heater can be attached and detached at any time without removing the inhaler from the patient's face or disturbing the anesthetic in any way. There is what might be termed a small rheostat a short distance from the heater for controlling the radiation.

METHODS OF ADMINISTRATION.

When ether is administered by the closed drop method the stand and tubing are not used, unless oxygen is administered to prevent anoxemia. Otherwise the air vent is left slightly open, enabling the patient to inhale a small amount of air with each inspiration from the bag, and the amount of rebreathing controlled by the proportion the air vent is opened. Ethyl chlorid is administered by the closed method by spraying this anesthetic into the bag through the air vent, or through the rubber stop-cock.

When ether, anesthol, ethyl chlorid and chloroform are administered by the open method the bag is disconnected, thus permitting of a free supply of air and no rebreathing. The administration of the anesthetic, excepting ethyl chlorid, is controlled by the needle-point valve. As the exhalations escape at the expiratory orifice, instead of passing over the gauze, much less of the anesthetic is used.

*Warm Vapor Method.*—For ether by the open method one of the two higher grades of heat is used; and for the other anesthetics and methods one of the two lower grades of heat is sufficient. The hand holding the mask to the patient's face constantly furnishes the information as to the temperature of the chamber. For ether by the open method, it should be kept as hot as the hand can well tolerate it (120-125 F.) and for all other use it should be at about 100 to 105 F., or just merely warm. There is no practical way of holding the mask to the patient's face except by thus placing the hand in intimate relation to the source of heat—a very practical, efficient and constantly acting safeguard.

*The Closed or Rebreathing Method.*—As the breathing-space of my apparatus is ample throughout, and the bag placed close to the patient's face, it throws no extra work on the respiratory process in rebreathing, as in the case in which the patient breathes back and forth through a long tube. With a patient breathing rapidly (about thirty respirations per minute under nitrous oxid) and expiring 500 c.c. into a mask and a 30-inch tube of 1,000 c.c. capacity, it is certainly evident that none, or but very little, of the expiration reaches the bag before the next inspiration takes place, and that this inspiration consists almost entirely of a mixture of the previous expiration and the contents of the mask and tube. Consequently the patient continually inspires a higher percentage of carbon dioxid and a lesser amount of the anesthetic than would be the case were the bag placed close to the patient's face. In other words, when the bag is close to the face there is a retention of a less amount of carbon dioxid with the same amount of rebreathing, or with the same amount of carbon dioxid retained there in a longer period of rebreathing. Therefore it is a matter of economy as well as a better scientific principle to dispense with the long tube and place the bag close to the patient's face,and an extended clinical experience with both methods clearly establishes this proposition.

Another and decided advantage of having the bag andan - esthetic chamber close to the patient's face is that it enables the anesthetist to watch the movements of the bag, the dropping of the anesthetic and the patient's face all at the same time. He can thus concentrate his attention on a small space.

In order to ascertain the temperature of the vapor inhaled when ether is administered by the open drop method, I placed a specially constructed and delicately calibrated thermometer within an inch of the gauze in the herein described inhaler—the approximate distance of the patient's respiratory passages from the gauze of the usual open mask—and, since the expirations did not influence the thermometer, this gave the temperature accurately of the inspired vapor. Sufficient ether for a light anesthesia quickly reduced the temperature to 55 F.; a moderate anesthesia to 45 F., a deep anesthesia to 35 F., and a profound anesthesia to 32 F., operating room temperature 75 F.

After determining the temperature of the cold vapor I attached the electric heater to the ether chamber and I found the temperature then to vary from 80 to 88 F. (average 84 F.) in a room temperature of 75 F. In other words, the electric heater raised the temperature of the vapor on an average 42° F. by the thermometer. But in this apparatus, after passing over the thermometer the vapor passes through 3 inches of a metal tube maintained at a temperature of about 110 F., which further elevates the temperature of the vapor several degrees, and, on account of a low specific heat of gases, the vapor was warmed to practically body temperature at the time it was inhaled.

### ADVANTAGES.

1. The design, construction and operation are reduced to the basic principles of simplicity.

2. Each and every one of the inhalation anesthetics can be administered by all the different methods in common use. It can be easily, quickly and thoroughly sterilized, and above all else it is practical.

3. Being compact in design, light in construction and easily assembled and taken apart, the entire apparatus, together with sufficient nitrous oxid and oxygen for two hours' anesthesia, can be readily carried in an ordinary handbag.

4. The whole apparatus is not expensive; any parts not wanted need not be purchased. As it covers the entire field for general anesthetics by the oral-nasal route, no other equip mentis needed except for the highly specialized methods. It saves one-third of the cost of all anesthetics administered by the open method, and reduces to an absolute minimum the cost of the anesthetic by the rebreathing method.

5. For nitrous oxid administration it furnishes a light and compact stand for holding the proper number of cylinders and places the rubber bag close to the patient's face. The stand is absolutely rigid with slip connections. It affords practical means for administering ether by a new method—the closed drop method, which is the logical and scientific complement of the open drop method. For ethyl chlorid administration by the open method, it insures that the anesthetic will always be completely vaporized and diluted before being inhaled. It thoroughly warms the vapor of ether by the open drop method. It always shows whether the patient is actually breathing—no matter what anesthetic is used or method employed. It warms the vapor of all anesthetics to practically body temperature at the time it is inhaled. It reduces the administration of ether to a scientific basis—any desired rate of administration can be obtained in the open, semi-open or closed methods and maintained with absolute constancy. In the open method it prevents the waste of the anesthetic from vaporization by the expirations. It furnishes a compact, light, convenient practical and scientific means for administering all the general anesthetics by all the ordinary methods. It affordsthe utmost possible conservation of the patient's energy.—*JournalA.M.A.*

## Figures and Tables

**Figure f1:**